# Mechanism of Staple Line Leak After Sleeve Gastrectomy via Isobaric Pressurisation Concentrating Stress Forces at the Proximal Staple Line

**DOI:** 10.1007/s11695-022-06110-z

**Published:** 2022-05-31

**Authors:** William Catchlove, Sam Liao, Gillian Lim, Wendy Brown, Paul Burton

**Affiliations:** 1 Department of Surgery, Central Clinical School, Monash University, Alfred Health Centre, Level 6, 99 Commercial Road, Melbourne, VIC 3002 Australia; 2 Oesophago-Gastric and Bariatric Surgery Unit, Alfred Hospital, Melbourne, VIC Australia; 3 Department of Mechanical and Aerospace Engineering, Monash University, Melbourne, VIC Australia

**Keywords:** Staple line leak, Leak, Sleeve gastrectomy, Complication, Finite element analysis, Bariatric surgery, Obesity, Intraluminal pressure, Oesophageal manometry, Gastric pressure

## Abstract

**Purpose:**

Staple line leak following sleeve gastrectomy is a significant problem and has been hypothesised to be related to hyperpressurisation in the proximal stomach. There is, however, little objective evidence demonstrating how these forces could be transmitted to the luminal wall. We aimed to define conditions in the proximal stomach and simulate the transmission of stress forces in the post-operative stomach using a finite element analysis (FEA).

**Materials and Methods:**

The manometry of fourteen patients post sleeve gastrectomy was compared to ten controls. Manometry, boundary conditions, and volumetric CT were integrated to develop six models. These models delineated luminal wall stress in the proximal stomach. Key features were then varied to establish the influence of each factor.

**Results:**

The sleeve gastrectomy cohort had a significantly higher peak intragastric isobaric pressures 31.58 ± 2.1 vs. 13.49 ± 1.3 mmHg (*p* = 0.0002). Regions of stress were clustered at the staple line near the GOJ, and peak stress was observed there in 67% of models. A uniform greater curvature did not fail or concentrate stress under maximal pressurisation. Geometric variation demonstrated that a larger triangulated apex increased stress by 17% (255 kPa versus 218 kPa), with a 37% increase at the GOJ (203kPA versus 148kPA). A wider incisura reduced stress at the GOJ by 9.9% (128 kPa versus 142 kPa).

**Conclusion:**

High pressure events can occur in the proximal stomach after sleeve gastrectomy. Simulations suggest that these events preferentially concentrate stress forces near the GOJ. This study simulates how high-pressure events could translate stress to the luminal wall and precipitate leak.

**Graphical Abstract:**

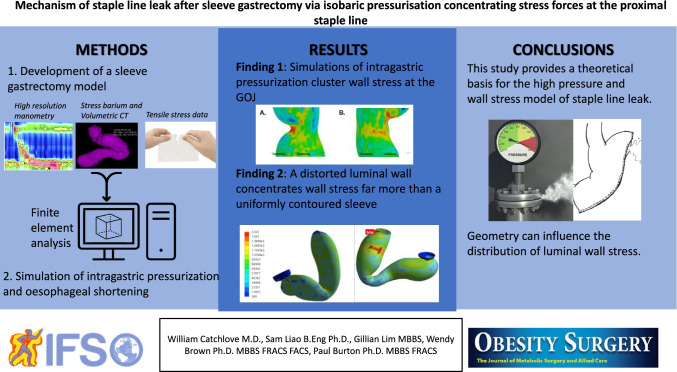

## Introduction

Staple line leak is a morbid complication after sleeve gastrectomy and frequently results in a protracted recovery, significant morbidity, and occasionally mortality [1, 2]. Leaks almost always occur in the proximal staple line within 1–2 cm of the gastro-oesophageal junction [3, 4]; however, the precise pathophysiology is not well delineated [5]. An understanding of the precipitating factors driving leak could provide technical insights into sleeve construction that could reduce the incidence of chronic leak and aid decision making during their management.

Various mechanisms have been proposed to be the driver of leak; however, no single factor has been determined to be the sole contributory factor. Ischaemia and hyperpressurisation of the proximal stomach have been proposed to be the two main causative factors for leak. In-flow hypoperfusion, and a paucity of vasculature in the proximal staple line, has been suggested to drive ischaemia in the post-operative period [6]. Other theories, including thermal injury to the gastric wall or compromise to vascular supply during dissection, have been postulated.

Evidences of high-pressure events in the stomach after sleeve gastrectomy have led to the hypothesis that they play a role in the initiation and perpetuation of sleeve leak [7–9]. There have been multiple studies investigating staple line burst pressures and buttressing methods in an effort to reduce the incidence of leak [10]; however, the transmission of stress forces to the luminal wall has not been examined at length.

We utilised the finite element method (FEM) in order to predict the behaviour of the post-operative stomach under specific biological conditions. FEM is a commonly used mathematical method to solve complex mechanical and biological problems using sophisticated computing [11, 12]. A larger structure is reduced into individual finite elements and their behaviour under specific conditions is solved during repeated simulation. Accurate simulation of events requires a precise understanding of anatomy, pressure events, and tissue strength.

Our hypothesis is that using finite element analysis (FEA), we can develop a model of the post-operative stomach and using specific boundary conditions, we will be able to simulate the transmission of stress forces to the luminal wall. This will give us insights into the influence of the geometry of the sleeve on stress forces.

## Methods

### Patient Selection

There were two components to this study. In part 1, we established boundary conditions to accurately inform our intended finite element analysis. This involved compiling intraluminal pressure and anatomical data. In part 2, we conducted finite element analysis under different conditions with boundary conditions informed with data from part 1.

### Part 1: Boundary Conditions

We utilised data from a prospective clinical trial from within our unit [13]. This involved patients who had undergone a sleeve gastrectomy from between 2014 and 2019. Patients were included if they were aged between 18 and 65 years, at least 6 weeks post-operative, and had an anatomically unremarkable gastric sleeve on contrast swallow. An unremarkable sleeve was defined as a tubular-shaped stomach without a retained fundus or evidence of obstruction or contrast leak. Patients were excluded if they had previous non-bariatric oesophago-gastric surgery or had a pre-existing oesophago-gastric motility disorder.

#### Surgical Technique

We performed a laparoscopic sleeve gastrectomy using a 36 French ViSiGi™ bougie and stapled with a tri-stapler (Echelon Flex ™ GST). Once the stomach was mobilised, stapling began 4 cm from the pylorus and finished at least 2 cm away from the GOJ. The staple line was imbricated with a running suture.

#### High Resolution Manometry

**Fig. 1:**
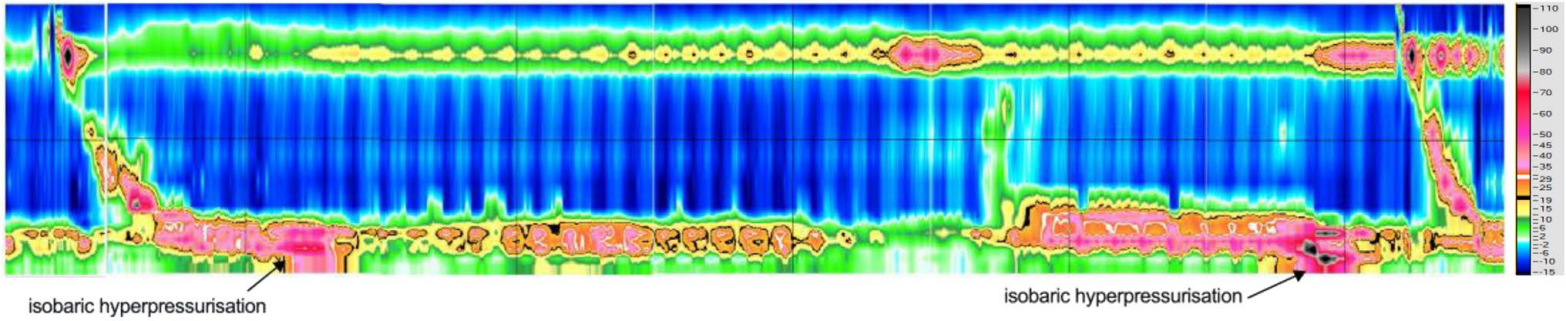
High resolution manometry demonstrating isobaric pressurizations after a swallow in a sleeve gastrectomy patient

A 16-channel manometry catheter, attached to a water perfused system, was inserted transnasally. The distal sensor was positioned in the proximal stomach 2 cm below the inferior border of the lower oesophageal sphincter (Fig. 1). Data were recorded in real time using TRACE!1.2 (written by G. Hebbard using LabVIEW, National Instruments, Austin, TX). Our technique has been previously described [9]. We used a standardised protocol for each patient: (1) supine basal recording for 60 s, (2) 5 deep breaths, (3) 10 swallows with 5 ml water, (4) volume stress test (5 swallows of 10 ml water). Basal pressure was defined as end expiratory pressure.

#### Stress Barium

A retrospective analysis of sleeve gastrectomy patients who had undergone a stress barium at our institution was undertaken. The technique for stress barium was adapted from a previous technique [14]. Two swallows of 5 mL liquid barium were taken, with anterior radiographs taken. This process was repeated for the lateral view. In order to distend the stomach, patients ingested two spoonsful of barium-soaked porridge followed by 80 mL of undiluted porridge.

#### Volumetric CT Analysis

We used a standardised procedure for each patient’s volumetric CT. Each patient was fasted for a minimum of 6 h prior to CT scan. Immediately prior to the scan, patients were provided a two-part oral contrast solution of X-EVESS™ (*MCI Forrest*, *West Footscray*, *Vic*, *Australia*) containing a CO2 producing mixture of sodium bicarbonate and citric acid.

### Part 2: Establishment of Finite Element Models

#### Finite Element Analysis

FEA simulation was undertaken using ANSYS Software (Fluent, ANSYS 18.0, PA, USA). Baseline gastric geometry was determined using parameters from volumetric CT. Anatomical features of importance were considered to be triangulated apex (‘dog-ear’), narrow incisura, tapered apex, and leak. Gastric geometry was included in the study based on the presence of the aforementioned key anatomical features which have previously been implicated in staple line leak such as stenosis in the proximal stomach and retained fundus.

Additional fixed parameters across each model included the following: 1 mm uniform wall thickness, internal pressure of 4000 Pa (~ 30 mmHg) in the proximal and distal compartment, and a fixed oesophagus and pyloric sphincter. Tensile strength data of fresh gastric tissue were extracted from previously published work [15]. The tensile ultimate strength was 0.67 MPa. We assumed a density of 1040 kg/m^3^ and Poisson ratio 0.3.

### Part 3: Simulation Conditions

**Fig. 2:**
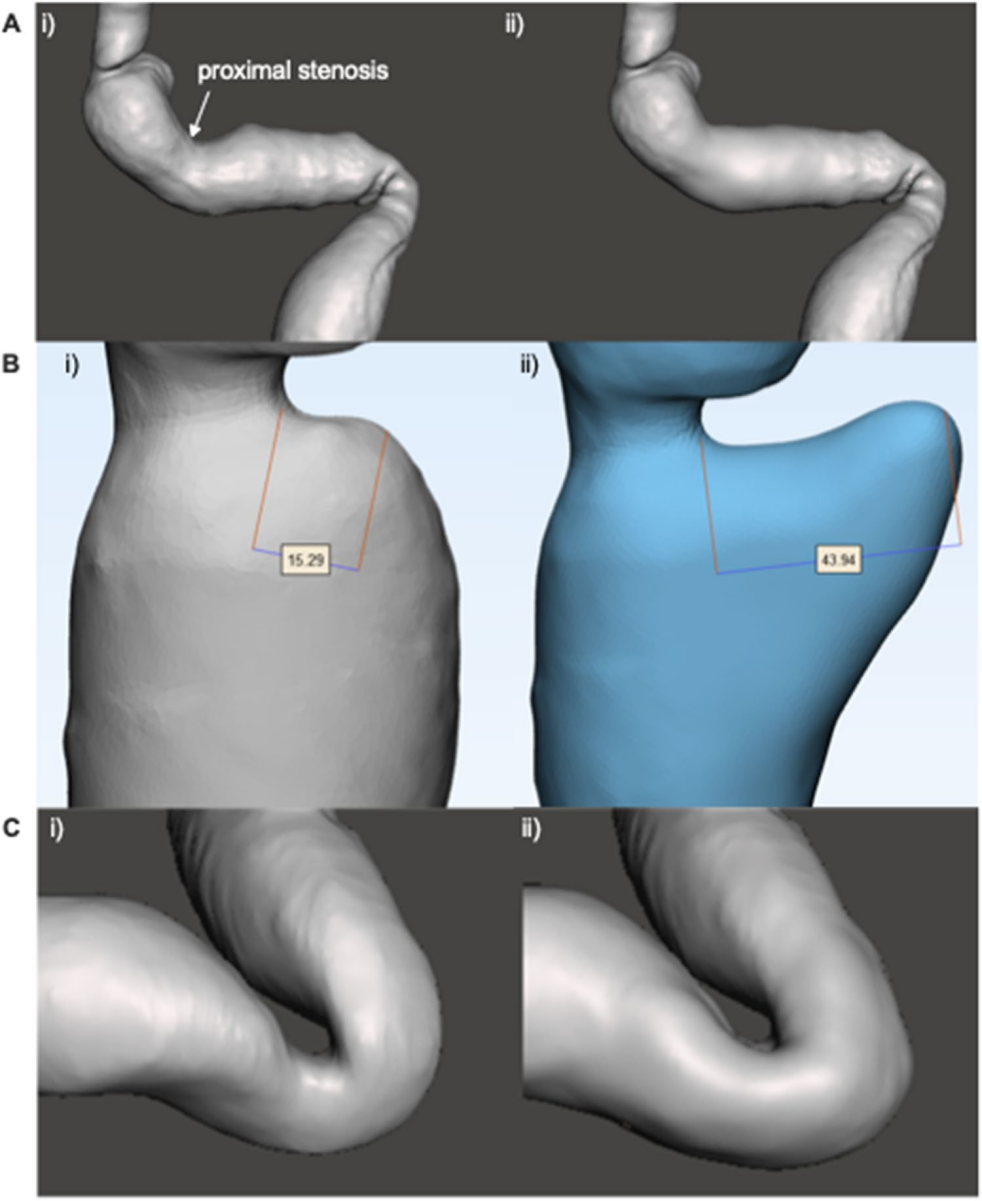
Variation in gastric geometry from the (i) original model to (ii) modified version. **A** proximal staple line stenosis, **B** triangulated apex, and **C** widened incisura

We simulated the conditions of a dry swallow by using longitudinal oesophageal displacement (shortening) of 3.5 cm during pressurisation [16]. The geometry of three models was also varied to evaluate the influence of key anatomical features on luminal wall stress (Fig. 2): proximal stenosis, triangulated apex, and the diameter of the incisura. A proximal stenosis was identified and dilated until it was a more consistent calibre (Fig. 2A). Similarly, a triangulated apex was created by enlarging a smaller apex (Fig. 2B), and the incisura of a model was widened from 42 to 50 Ch (Fig. 2C).

### Statistical Analysis

All statistical analysis was performed with GraphPad Prism version 9.2.0 (GraphPad Software, San Diego, CA, USA). Continuous parametric data were analysed using Students *t* test. Where comparisons of repeated measures within the same subject were used, we used a repeated measures ANOVA. A *p* value < 0.05 was considered significant. Data were presented as mean ± standard deviation.

## Results

### Part 1: Establishment of Boundary Conditions

**Fig. 3:**
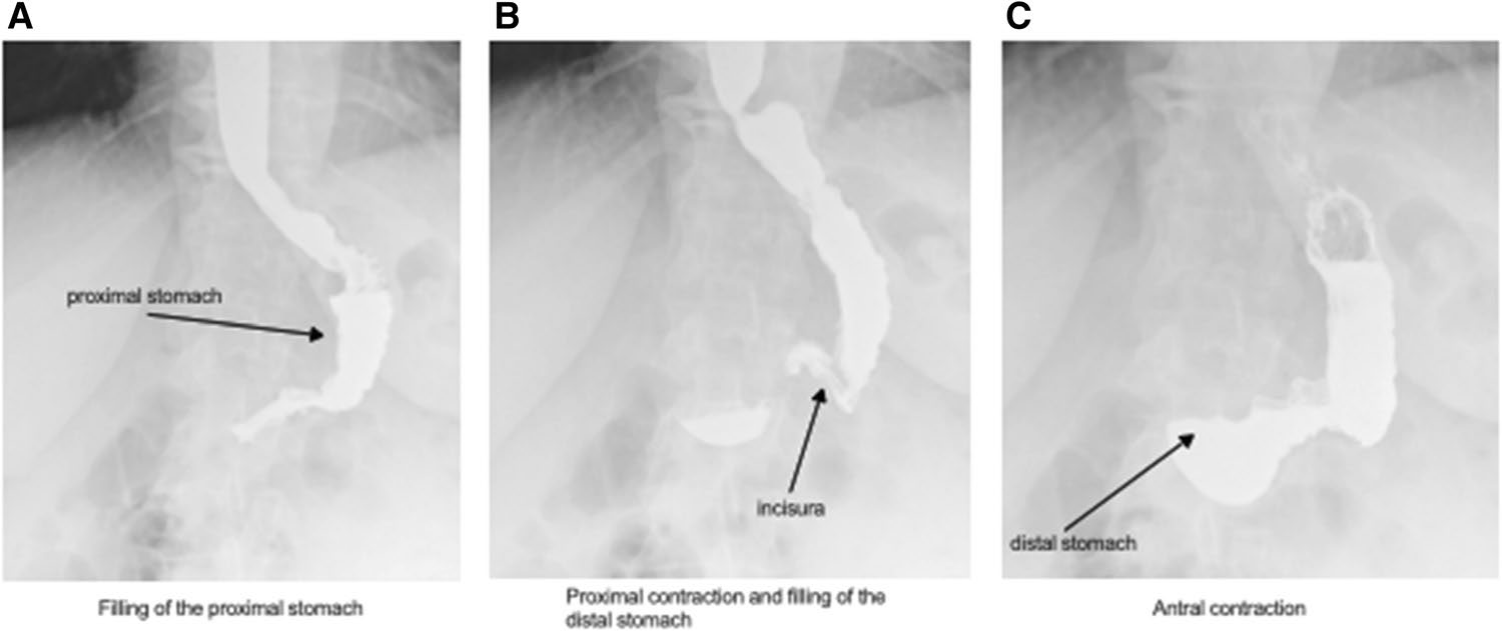
Stress barium after sleeve gastrectomy with bicompartmental sleeve demonstrating **A** filling of the proximal stomach, **B** proximal contraction and filling of the distal stomach, and subsequent **C** antral contraction

**Fig. 4:**
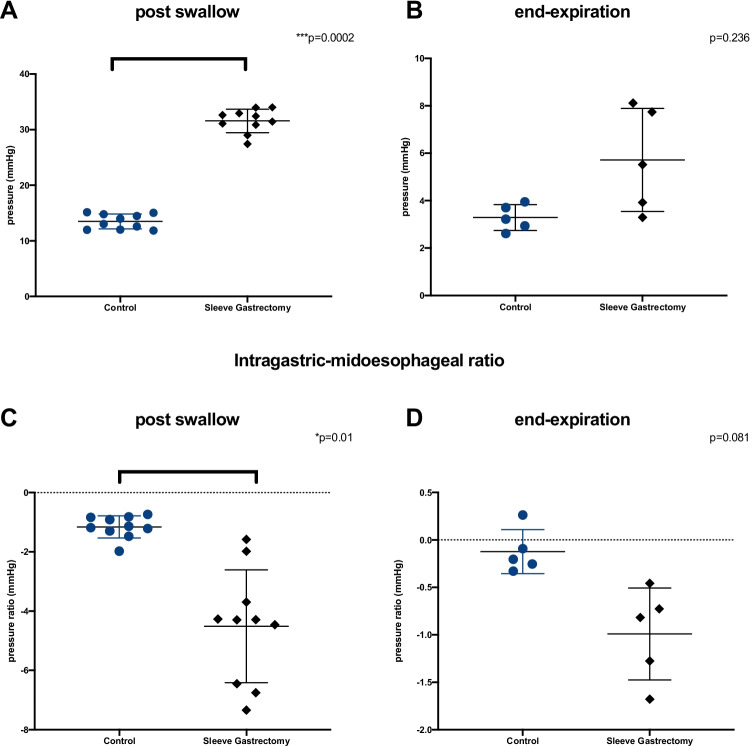
Evaluation of high-resolution manometry. Peak intragastric isobaric pressures measured at **A** immediately after a normally structured wet swallow of 5 ml of water, and **B** at end expiration during the basal recording period. Intragastric/mid-oesophageal ratio of **C** peak intragastric pressure during a normally structured swallow and **D** at end expiration during the basal recording period

i. **Barium Swallow**
  Barium swallow studies after a sleeve gastrectomy appear to suggest a bicompartmental appearance of the post-operative stomach (Fig. 3). Swallowing is characterised by the filling of the proximal compartment, subsequent pressurisation, and emptying into the distal compartment. We surmised that previously reported intragastric hyper-pressurisations were related to this distention of the proximal compartment as this has been noted during concurrent fluoroscopy and intraluminal pressure measurements [9].
ii. **Measurement of Intraluminal Pressure in the Fasting State (Prolonged Study)**
  In order to establish the range of intragastric pressurisation, we assessed the high-resolution manometry (HRM) of fourteen patients after sleeve gastrectomy and compared then to ten obese controls. The results are described in Table 1. The median time between sleeve gastrectomy and manometry was 6 months (range: 5–31). The immediate post-operative period was uncomplicated for all these patients, and none developed staple line leak post operatively. Twelve of these patients underwent HRM for investigation of new reflux symptoms post operatively; the remaining two patients report new symptoms of dysphagia after their operation.
  Ten obese control patients underwent manometry as a preoperative investigation prior to bariatric surgery. The demographic details of the control and sleeve gastrectomy groups were respectively: age 40.5 ± 17.7 vs 42.5 ± 15.7 (*p* = 0.22), gender 9 (90%) vs 11 (78.5%) female (*p* = 0.615), weight 122.3 ± 21.0 kg vs 110.6 ± 18.9 kg (*p* = 0.771), BMI 48 ± 11.7 vs 38.3 ± 3.8 (*p* = 0.023). BMI was significantly lower in the sleeve gastrectomy cohort (*p* = 0.023).
  The sleeve gastrectomy cohort had a significantly higher peak intragastric isobaric pressures after normally structured swallows than those in the obese control group 31.58 ± 2.1 vs. 13.49 ± 1.3 mmHg (*p* = 0.0002) (Fig. 4). The IG/MO ratio at each point of hyperpressurisation was more negative after a swallow in the sleeve gastrectomy patients −4.36 ± 7.4 versus −1.17 ± 1.4 mmHg (*p* = 0.01). There was no significant difference between intragastric pressure in sleeve gastrectomy patients who had hiatus hernias 35.3 ± 11.3 mmHg versus 28.8 ± 8.4 mmHg in those without (*p* = 0.435).
  During the basal stage of monitoring, there was no significant difference in peak pressures at end expiration 3.28 ± 3.4 vs. 5.78 ± 6.5 mmHg (*p* = 0.236). Similarly, there was no difference in the IG/MO ratio during the basal phase −0.123 ± 0.8 vs. −1.0 ± 1.8 mmHg (*p* = 0.081) (Fig. 4).
  We delineated using HRM that the sleeve demonstrated isobaric pressurisations relating to distension of the proximal compartment. This led us to construct a model in which the distended pressurised state would exist even when swallowing minimal volumes.

**Table 1:** Demographic and clinical outcomes of HRM

	Obese control	Sleeve gastrectomy	*p* value
Patients (*n*)	10	14	-
Female (%)	90%	78.5%	0.615
Age (years)	40.5 ± 17.7	42.5 ± 15.7	0.220
Weight (kg)^†^	122.3 ± 21.0	110.6 ± 18.9	0.771
BMI (kg/m^2^)^†^	48 ± 11.7	38.3 ± 3.8	0.023
LOS relaxation	10 (100%)	13 (92.9%)	0.99
Oesophageal peristalsis	10 (100%)	13 (92.9%)	0.99
Hiatus hernia	2 (20%)	7 (50%)	0.209
Motility disorder	2 (20%)	2 (14.3%)	0.99
Abnormal peristalsis observed	5 (50%)	5 (35.7%)	0.678
Repeat swallows observed	10 (100%)	14 (100%)	-
Peak isobaric proximal intragastric pressure (mmHg)	13.49 ± 1.3	31.58 ± 2.1	0.0002
Isobaric intragastric/mid-oesophageal pressure ratio (mmHg)	− 1.17 ± 1.4	− 4.36 ± 7.4	0.01
Basal intragastric pressure (mmHg)	5.78 ± 6.5	3.28 ± 3.4	0.236
Separation of LES from diaphragm (cm)*	0 (0–3)	0 (0–2)	0.99

^*^Median (range), ^†^at time of manometry

### Part 2: Model Geometry Using Volumetric CT

The volumetric CT of 22 patients were assessed post sleeve gastrectomy. The median time after surgery was 2 years (range: 6 months–12 years).

Of the 22, 21 patients (95.5%) had a bicompartmental appearance. Four patients had a smoothly contoured proximal stomach. The remaining 18 patients had features of irregular contouring along the greater curvature of the proximal compartment. This included 2 patients with a tapered GOJ, 5 with a triangulated apex, 4 with a proximal stenosis, and 5 with a narrow incisura. One patient had a leak, which was known prior to the study.

We established that the post-operative stomach has a varied appearance which has implications for the transmission of mechanical forces to the gastric wall.

### Part 3: Simulation of Model Using Finite Element Analysis (FEA)

**Fig. 5:**
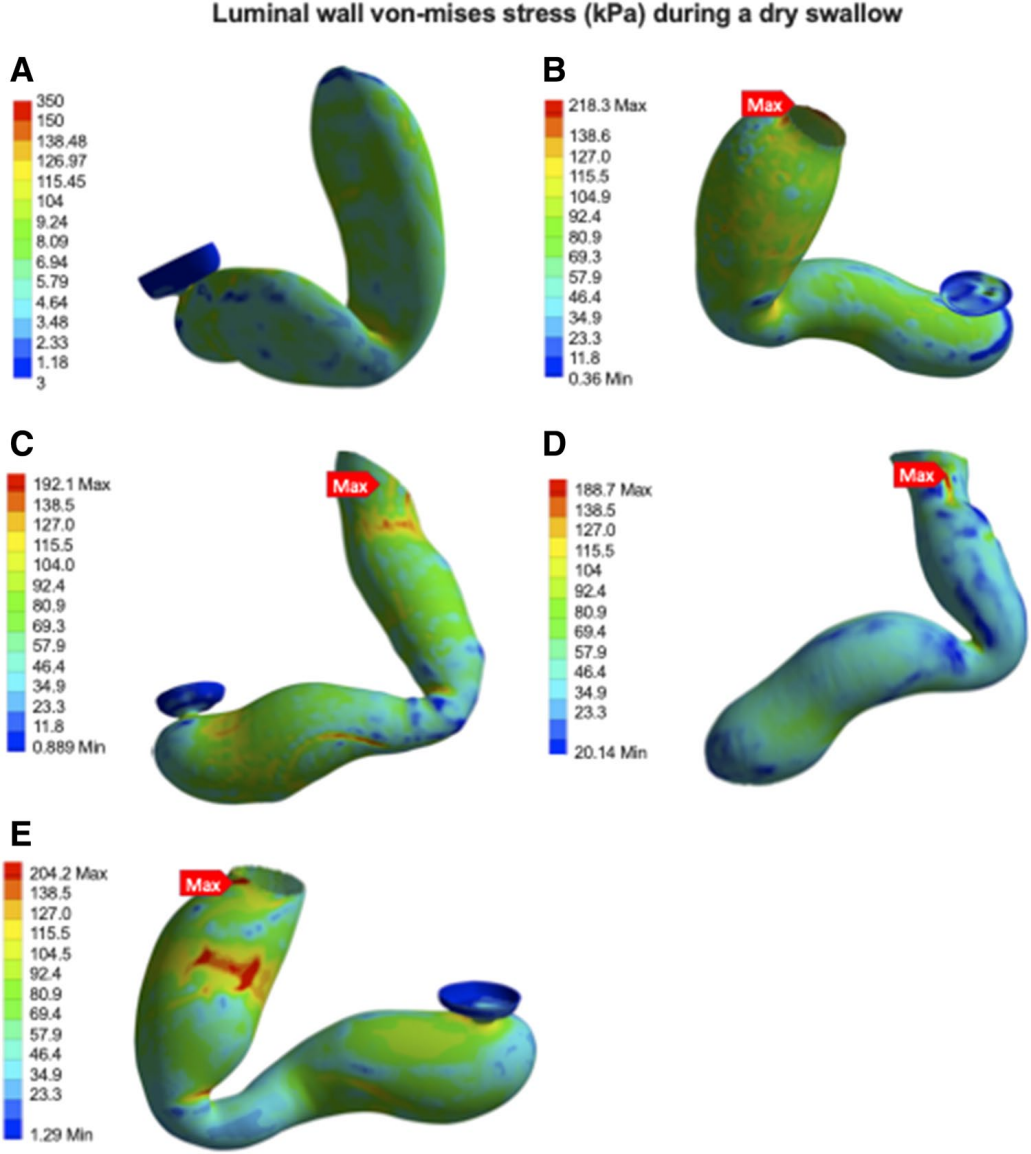
Luminal wall (von-mises) stress of the stomach during a simulated dry swallow with maximal stress and site of tissue failure marked (**A**–**E**). **A** Anterior view of smoothly contoured greater curvature model without evidence of tissue failure during simulation. **B** Posterior view of small dog-ear model with maximal wall stress acting on the GOJ at 218 kPa. **C** Anterior view of model with proximal stenosis with maximal wall stress acting on the posterior GOJ of 192 kPa. **D** Anterior view of narrow incisura model demonstrating peak wall stress of 189 kPa at the staple line at the GOJ. **E** Posterior view of tapered apex model demonstrating peak wall stress of 204 kPA at the proximal staple line near the GOJ. There is a cluster stress forces at the site of a posterior indentation

**Fig. 6:**
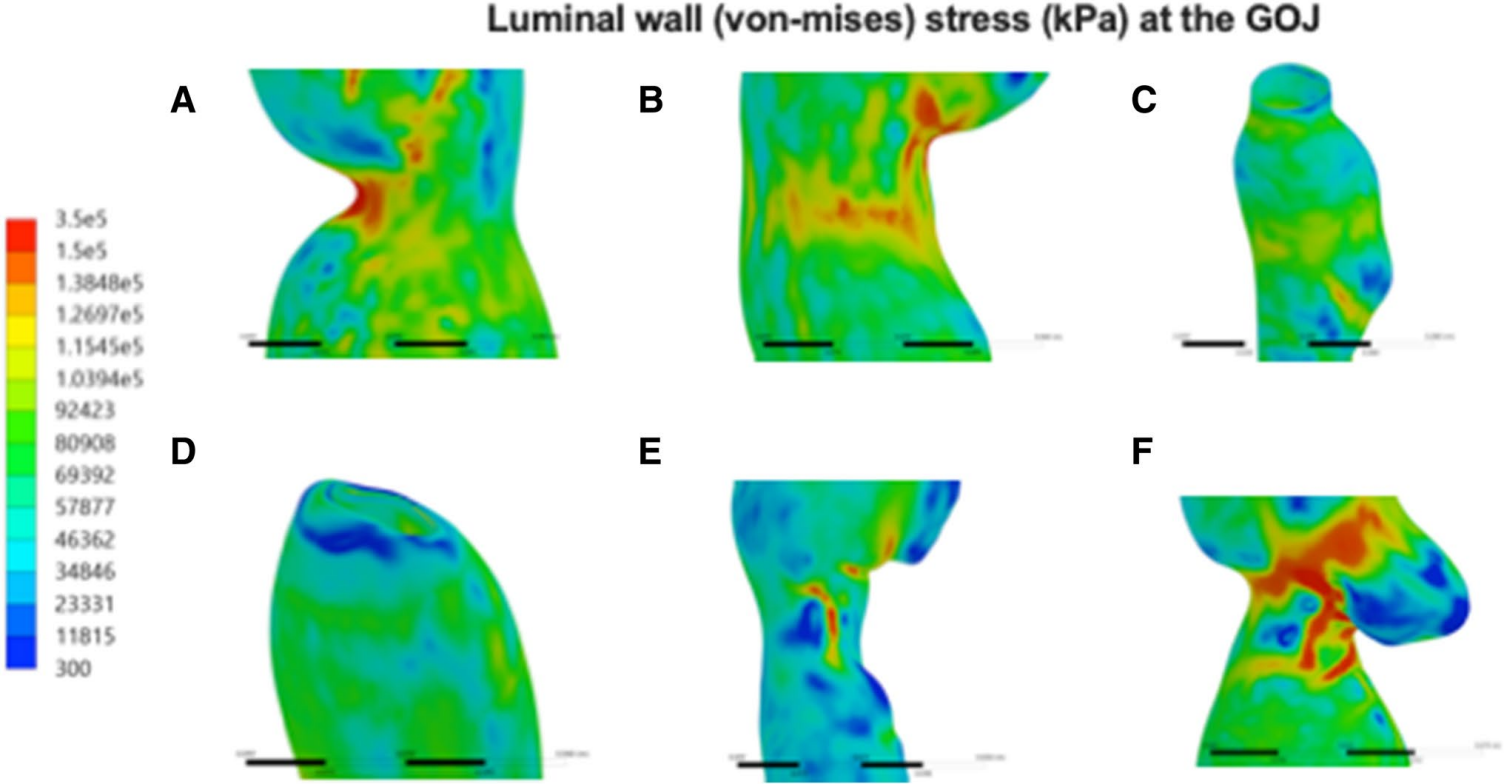
Simulation of luminal wall (von-mises) stress (kPa) at the GOJ and in the distal oesophagus of models from **A** small triangulated apex, **B** proximal stenosis, **C** leak, **D** smooth contour, **E** narrow incisura, and **F** tapered apex. Simulations demonstrate peak stress at the GOJ in models (**A**), (**B**), (**E**), and (**F**)

**Fig. 7:**
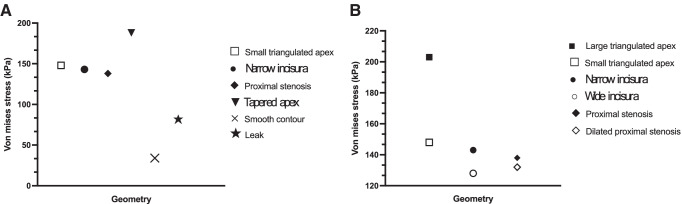
Peak equivalent stress at the GOJ during simulation. **A** Comparison between the leaking and the intact gastric models and **B** before and after variations in geometry at the greater and lesser curvature

**Fig. 8:**
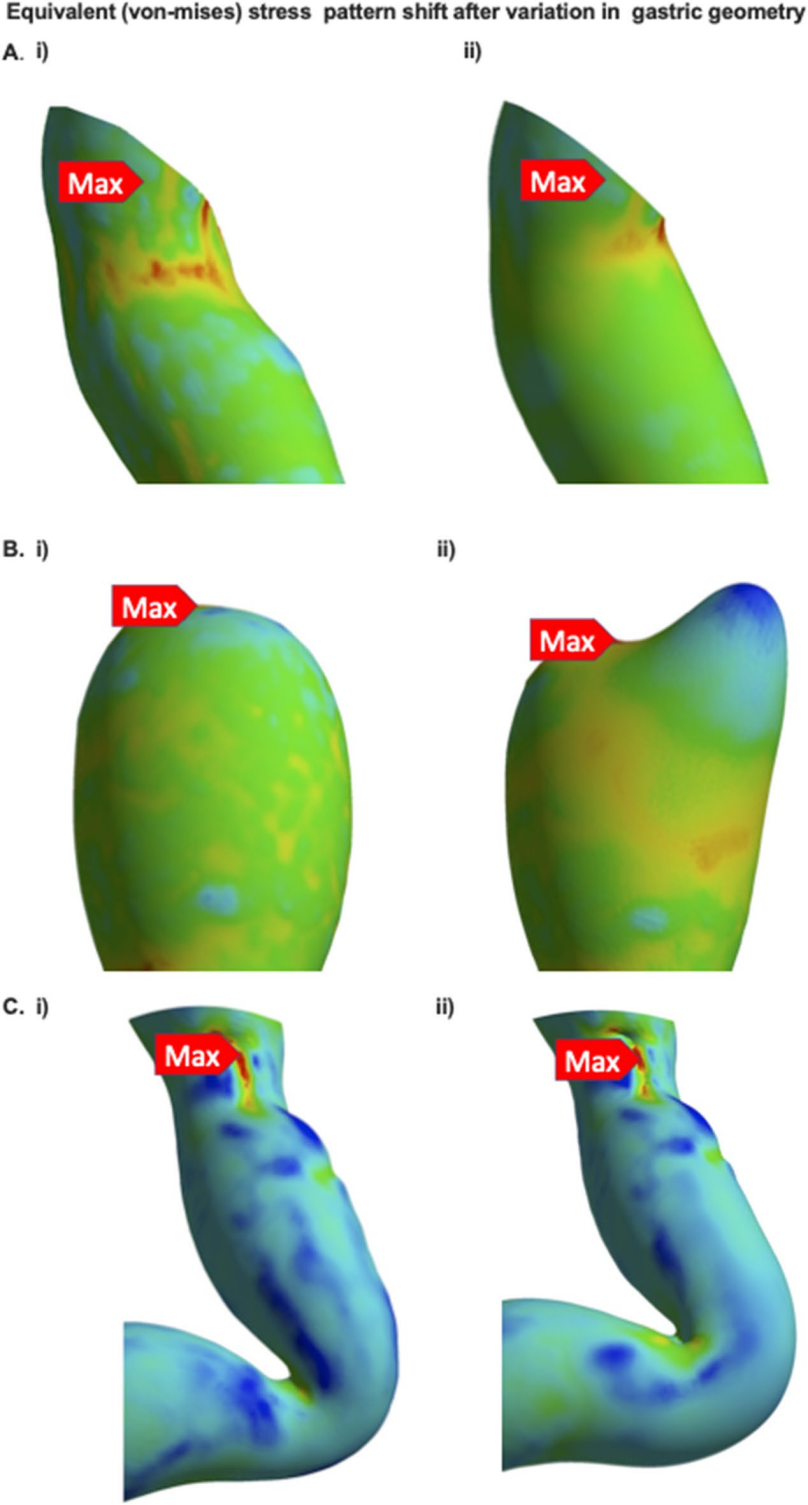
The original (i) and modified (ii) models of sleeved stomachs undergoing simulation of a dry swallow. **A** Dilation of the proximal stenosis. **B** Enlargement of the triangulated apex to be 4 cm from the GOJ. **C** Widening of the incisura from 42 to 50 Ch in the short axis

**Fig. 9:**
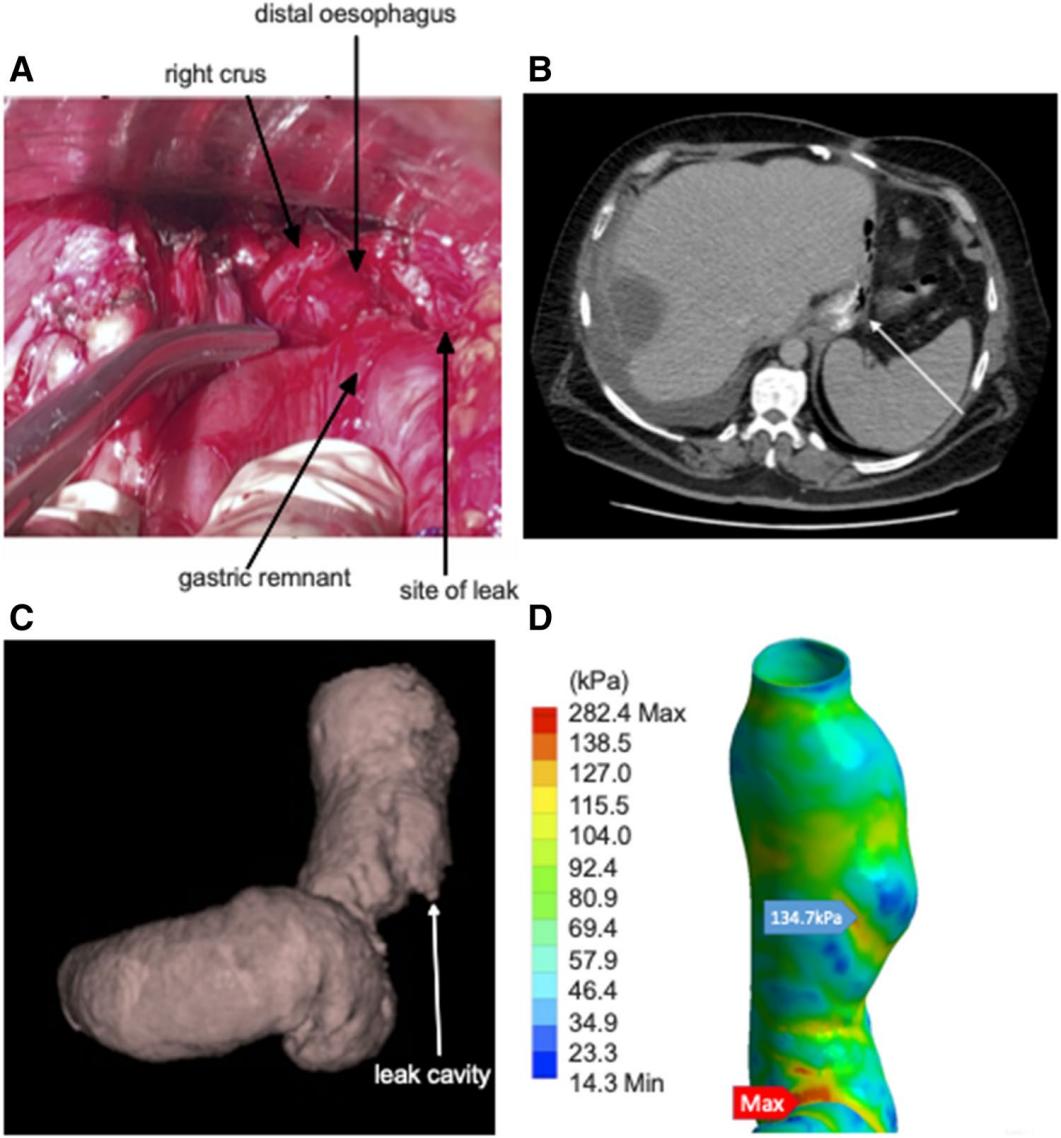
Finite element model of proximal staple line leak. **A** Total gastrectomy and oesophagojejunostomy with the site of the leak cicatrizing the stomach. **B** Axial section of abdominal CT with extraluminal gas (arrow) tracking through fistula. **C** Rendered stomach from the volumetric CT taken of a patient with staple line leak after sleeve gastrectomy demonstrating leak cavity (arrow). **D** FEA simulation with maximal wall stress at the incisura of 282 kPa. Peak stress at the site of leak was 135 kPa

i. **Simulation of a Dry Swallow**
  From the information delineated in parts 1 and 2, five distinct models of post-operative stomachs were established and underwent finite element analysis (Fig. 5). These models underwent a simulation of a dry swallow with oesophageal shortening and uniform pressurisation. During these simulations, we were able to determine luminal wall stress on different components of these models.
  All simulations showed lower stress in the distal stomach wall compared to the proximal stomach. Stress forces were also found to be clustered at staple line adjacent to the GOJ, and maximal wall stress was observed there in the majority of models (Fig. 6). Only one model, with a uniformly contoured greater curvature, did not show concentrated deformation or material failure under maximal pressurisation (Fig. 5A). The highest stress geometry was the small triangulated apex model (Fig. 5B) at 218 kPa. The uniformly contoured model had the lowest stress at the GOJ with 34 kPa (Fig. 7A). GOJ stress in the tapered apex simulation (Fig. 6F) had the highest stress along the staple line at the GOJ (188 kPa) (Fig. 7A).
ii. **Variation in Geometry**
  Three of the existing models (Fig. 5B–D) were then modified to assess the effect of variation in geometry on the stress patterns acting on the gastric wall (Fig. 8).
  Stress distribution was more uniform in the model with a dilated proximal stenosis (Fig. 8A i–ii). Von-mises stress was found be similar in both models, with a 4% reduction in peak stress in the modified simulation (192 kPa versus 185 kPa respectively). The larger triangulated apex model had a 17% higher maximal stress compared to the smaller triangulated apex model (255 kPa versus 218 kPa respectively) (Fig. 7B i–ii). Widening of the incisura did not result in a shift of stress forces but resulted in a 2.7% reduction in maximal wall stress (188 kPa versus 183 kPa respectively) (Fig. 8C i–ii).
  The large triangulated apex model had a 37% increase in peak stress at the GOJ compared to the smaller triangulated apex model (203 kPA versus 148 kPA) (Fig. 7B).
  There was a 4.5% decrease in stress at the GOJ in the dilated proximal stenosis compared to the unmodified model (132 kPa versus 138 kPa). A wider incisura led to a 9.9% reduction in stress at the GOJ during simulation (128 kPa versus 142 kPa).
iii. **Simulation of the Leak Model**
  A simulation of a swallow after staple line leak showed the highest stress forces around the incisura at 282 kPa (Fig. 9). This was more than twice the stress simulated at the leak site (135 kPa). The peak wall stress at the site of leak was lower than each other simulated geometry, except for the smoothly contoured sleeve. Similarly, equivalent stress at the GOJ was lower in the leak model, except when compared to the smooth contoured sleeve (Fig. 7A).

## Discussion

This study shows that there are significant isobaric high-pressure episodes that occur in the sleeve during oesophageal contractions. We have described how maximal shear forces could be transmitted to the staple line and precipitate tissue failure. Simulations of high-pressure events predict how maximal strain could be transmitted to the staple line at the GOJ during these events.

The distribution of luminal wall stress can be influenced by the geometry of the greater curvature. A smoother staple line is less likely to concentrate stress forces during simulation. An enlarged triangulated apex appeared to be a higher stress geometry during simulation of the key variable features of construction.

In our simulations the smoothly contoured greater curvature did not concentrate stress forces at any point along the staple line, suggesting that high stress events are not intrinsic in the construction of a sleeve gastrectomy. This may be a key factor in mitigating the risk of protracted staple line leak. In contrast, simulations suggest that the leak creates a lower wall stress geometry. This is consistent with the paradigm of the high-pressure sleeve, suggesting that leak has a decompressive effect.

The decline in incidence of staple line leak has been made more notable by the rise in popularity of the sleeve gastrectomy procedure. The reported incidence of leak varies between surgeons and institution, however, is approximately between 0.2 and 4% [5, 17]. The decrease in incidence has been attributed in-part to advances in surgical technique [18] and technology [19].

Intragastric hyper-pressurisation has been previously described after sleeve gastrectomy [9, 20]; however, there have been few studies demonstrating how these forces could be translated to the luminal wall and precipitate leak. Mid-gastric stenosis or incisura stricture has been suggested to play a role in leak [5]; however, not all cases of leak have evidence of stenosis [21, 22]. Parikh et al.’s meta-analysis found that a larger bougie size was associated with a reduction the risk of leak, suggesting that there is an intraluminal mechanism driving leak [23]. No other studies have utilised finite element analysis of gastric geometry to evaluate the transmission of stress forces along the staple line, despite its widespread application in biomechanics.

Marie et al.’s [24] porcine study describes a preferential burst pattern in the proximal sleeve after insufflation. Natoudi et al. found that similar burst pressures were required in the resected stomach after a sleeve [25]. Burst pressures in resected sleeve specimens have also been observed in our HRM findings [26]; however, these do not account for the geometry and dynamic shortening of the oesophagus during normal physiology. While the pattern of pressurisation is likely to differ in vivo due to various anatomical and physiological factors, the isobaric hyper-pressurisations found during our study were significant enough to theoretically cause gastric tissue failure using these findings. Associations between a thinner fundal wall and leak, while not significant [27], correlate with the mechanical hypothesis of leak.

Impaired vascular supply has been proposed and implicated in staple line leak [5, 6] and is likely to be associated in some leaks, but there is limited objective evidence to suggest that it is the major precipitating factor driving leak. Preliminary angiography findings published by Furia et al. on a small cohort of patients found limited evidence of vascular compromise after sleeve [28]. Despite these findings, it is possible that perfusion may play a role in multifactorial leak, but it does not appear to be the main causative factor. Studies assessing the association between leak and technical choices are difficult to adequately power given varied technique and relatively low incidence of leak, but suggest that there is no strong association between the two variables [19].

The strength of our study is that it uses sophisticated computer modelling to simulate the transmission of stress forces using real world tensile stress data during swallowing. We developed precise boundary conditions in our model to incorporate realistic conditions during swallow events and pressurisation. Simulation allows us to understand the influence of variation in sleeve construction without having to utilise large sample sizes or control for independent variables.

While FEA allows for flexibility in repeated simulation in a multitude of different conditions, it is difficult to account all of the physiological and biological factors influencing leak. Strict boundary conditions are necessary to create a functional model, but also remove the influence of independent variables. This model does not account for gastric contraction during filling and emptying of the stomach.

Future studies could use in vivo animal models to establish the influence of a particular geometry to precipitate leak; this will determine which factors need to be avoided to reduce the incidence of intractable leak. Further simulation using additional factors such intrinsic muscle contractility may provide more insight into the influence of geometry and the bicompartmental sleeve on gastric pressurisation.

## Conclusions

This study provides evidence of how high-pressure events in the proximal stomach can transmit stress forces preferentially to the luminal wall of the proximal stomach. It demonstrates how a smoothly contoured sleeve evenly distribute stress forces in the proximal stomach, and how the geometry of the sleeve can influence the distribution of these stress forces. It is possible that imbrication or other methods to vary the geometry after stapling may influence the distribution of stress forces. These findings provide evidence for the theoretical and scientific basis of the luminal wall stress model of staple line leak.
